# Assessing the diagnostic accuracy of the identification of hyperkinetic disorders following the introduction of government guidelines in England

**DOI:** 10.1186/1753-2000-2-32

**Published:** 2008-11-04

**Authors:** David M Foreman, Tamsin Ford

**Affiliations:** 1 Department of Child and Adolescent Psychiatry, Institute of Psychiatry at the Maudsley, King's College London, De Crespigny Park, London, SE5 8AF, UK; 2 Department of Health and Social Services, Isle of Man; 3 Peninsula College of Medicine and Dentistry, John Bull Building, Tamar Science Park, Research Way, Plymouth, PL6 8BU, UK

## Abstract

**Background:**

Previous studies have suggested that both underdiagnosis and overdiagnosis routinely occur in ADHD and hyperkinesis (hyperkinetic disorders). England has introduced governmental guidelines for these disorders' detection and treatment, but there has been no study on clinical diagnostic accuracy under such a regime.

**Methods:**

All open cases in three Child and Adolescent Mental Health Services (CAMHS) in the South East of England were assessed for accuracy in the detection of hyperkinetic disorders, using a two-stage process employing the Strengths and Difficulties Questionnaire (SDQ) for screening, with the cut-off between "unlikely" and "possible" as the threshold for identification, and the Development And Well-Being Assessment (DAWBA) as a valid and reliable standard.

**Results:**

502 cases were collected. Their mean age 11 years (std dev 3 y); 59% were clinically diagnosed as having a hyperkinetic disorder including ADHD. Clinicians had missed two diagnoses of hyperkinesis and six of ADHD. The only 'false positive' case was one that had become asymptomatic on appropriate treatment.

**Conclusion:**

The identification of children with hyperkinetic disorders by three ordinary English CAMHS teams appears now to be generally consistent with that of a validated, standardised assessment. It seems likely that this reflects the impact of Governmental guidelines, which could therefore be an appropriate tool to ensure consistent accurate diagnosis internationally.

## Background

Disorders involving attention, overactivity and impulsivity (hyperkinetic disorders) are now recognised as the commonest neurodevelopmental presentation in childhood [1]. Despite this, and the availability of effective treatments [2] there is lack of clarity over detection and diagnosis. The diagnostic systems of ICD-10 [3] and DSM IV [4] employ different diagnostic criteria, defining Hyperkinesis and Attention Deficit Hyperactivity Disorder (ADHD) respectively. The United States (US) and other countries that primarily use DSM IV report variability in detection that suggests both overdetection and underdetection, measured either directly or through using stimulant medication prescription as a proxy [5-8]; the United Kingdom (UK), which primarily uses ICD-10, reports underdetection only [9-11] despite contemporaneous international professional guidelines [e.g., [12]].

In England since 2000, the Government has intervened in this controversy by introducing practice guidelines for the detection of hyperkinetic disorders by the National Health Service (NHS) in addition to those provided by professional bodies, focussing primarily on secondary care [13], but there has been no investigation of diagnostic accuracy since their introduction. Accordingly, we assessed secondary care clinical diagnoses of ADHD and hyperkinesis against the standard set by the Development And Well-Being Assessment (DAWBA) [14], a well-validated instrument which was employed in the UK National Statistics surveys of child psychiatric morbidity [11,15].

## Methods

### Participant selection

East Berkshire is served by three secondary care Child and Adolescent Mental Health Service (CAMHS) teams, covering a total child (0–16) population of approximately 85,000. Each team had identical referral policies within the specified age-range, and diagnosed children according to NICE guidelines, which included assessment in multiple domains supported by questionnaires. Both ICD-10 and DSM IV diagnoses were used by all teams. All teams used the Strengths and Difficulties Questionnaire (SDQ) [16], as the teams are part of the CAMHS Outcome Research Consortium (CORC) [17]. The SDQ provides a probabilistic assessment of the likelihood of hyperkinetic disorders, based on UK population norms. Assessment policies differed slightly between the teams: one team routinely screened all referrals using the SDQ as a preliminary assessment of psychopathology; the other two teams employed the same questionnaire to detect ADHD before clinic assessment, if hyperkinetic disorders were suspected from the referral letter. Thus, in one team the SDQ informed all diagnoses made in the team, but in the other two the SDQ only informed the diagnoses of cases already suspected of having ADHD. Between October 2004 and July 2005 all cases from each team were reviewed, and included if: an assessment had been completed; the case was currently open to the team; and there was recorded evidence of activity in the case-file in the preceding 12 months. The child (0–16) community population served by each clinic was also enumerated, to allow estimation of predicted community prevalence as an indicator of sample representativeness.

### Reference standard & clinical diagnoses

The standard for ADHD diagnosis was that of the Development and Well-Being Assessment (DAWBA) [14,18], which had both sufficient validity and reliability, and two additional advantages for this study. First, the SDQ is an integral part of the DAWBA (providing an initial screen for caseness and diagnostic type), and so can be used for screening in the context of ordinary clinic activity; secondly, the DAWBA is the instrument employed by the National Statistics Mental Health of Children survey [11] and so ensures a close relationship with nationally accepted assessments. The DAWBA generates both ICD-10 and DSM IV diagnoses of hyperkinetic disorders. The DAWBA consists of highly structured questions closely related to the diagnostic criteria in both ICD-10 and DSM IV, supplemented with descriptions of problem areas in the informant's own words (parent, teacher or young person if aged 11 plus). A series of prompts explored these problem areas. Data from all informants and both the structured and qualitative parts of the DAWBA can be combined by trained clinical raters to assign diagnoses. Alternatively the data from the structured questions provides computer predictions about the likelihood of diagnoses based on data from several large national surveys that used the DAWBA (refs). DMF was the trained rater, having previously trained and rated cases on one of the national surveys. DMF trained SD, a psychology graduate, as the interviewer. DMF was blind to all other case-related data (i.e., clinic diagnosis and case-note information) when making ratings. As clinic notes frequently made no mention of the diagnostic system used in making the diagnosis, a single category of "hyperkinetic disorders" was used to identify all clinical diagnoses made. Clinical case-note diagnoses were coded by SD into six categories: hyperkinetic disorders; emotional disorders; non-hyperactive behaviour disorders; mixed disorders of behaviour and emotions; other disorders; and no disorder.

### Data collection

SDQ scores from all cases were collected; if a SDQ was not available from the file one was requested from the parents. If multiple SDQ informants were available, their scores were combined to produce the prediction; otherwise single SDQ scores, from either parent or teacher, were used. The earliest SDQ was used, if collection had occurred at several time points. As all teams used SDQs as the preliminary screen for hyperkinetic disorders, this protocol ensured that (except for cases transferred from elsewhere, already diagnosed) the SDQ used in the study was collected prior to clinical diagnosis of a hyperkinetic disorder. The resulting SDQ predictions for hyperkinetic disorders were compared with case-note files by SD. Cases were classified as concordant or discordant for hyperkinetic disorders according to table 1.

**Table 1:** Classification of agreement between strengths and difficulties questionnaire (SDQ) and case-note assessment of open cases†

**Case-note assessment**	**SDQ prediction**	**Hyperkinetic disorders**		
		**Unlikely**	**Possible**	**Probable**
**Hyperkinetic disorders identified**		Discordant	Concordant	Concordant
Hyperkinetic disorders* not identified		Concordant	Discordant	Discordant

*includes uncertain cases

**Figure 1:**
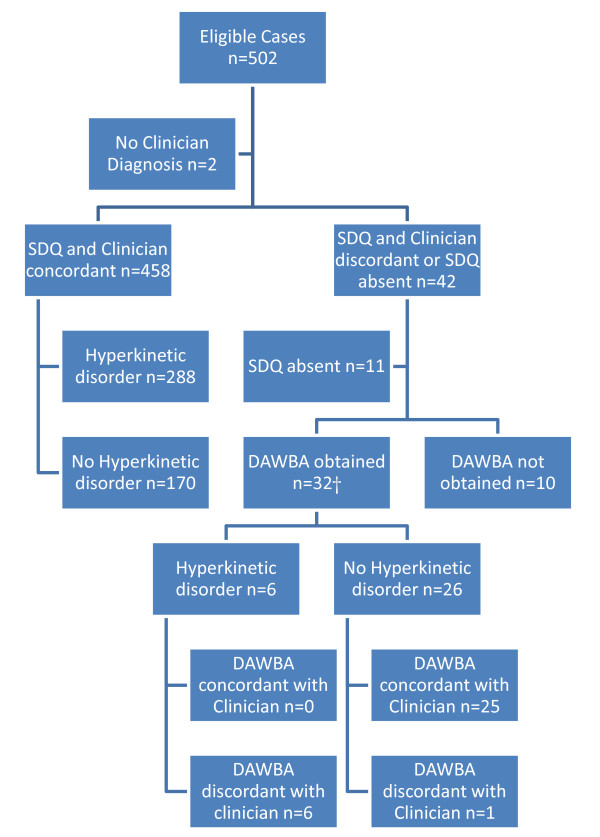
**Flow chart of recruitment and assignation of patients.** †includes 1 discordant SDQ collected at DAWBA interview.

† See figure 1 for numbers of classified cases

The cut-offs for discordancy chosen were based on the "unlikely" SDQ diagnostic prediction for hyperkinetic disorders being associated with a complete absence of such cases in its validation study [19], while a similar absence of clinical over-diagnosis with respect to the DAWBA was found in the ONS child psychiatric morbidity study [11]. All discordant case identified were invited for interview using the DAWBA, as were cases where previous attempts to obtain an SDQ had been unsuccessful, in a final attempt to obtain SDQ scores.

In routine assessment, clinicians would routinely seek confirmation of the pervasiveness of difficulties from teachers before making a diagnosis of ADHD or hyperkinetic disorder. However, if not previously present in the file, teacher-rated SDQs could not be obtained as permission to contact school was not routinely available; DAWBAs were likewise limited to parent interviews only.

### Ethics

On submission to the Local Resarch Ethics Committee, it was determined that the study should be managed under local audit protocols. However, it was agreed with the Local Research Ethics Committee that any discordant cases, where the DAWBA result disagreed with the clinician, would be fed back to the patient's clinician, who would have responsibility for discussing the finding with the patient and their family.

### Analysis

Diagnostic concordance between clinic diagnoses and the DAWBA were explored by descriptive statistics and cross-tabulations (see below); these analyses were conducted within the R statistical environment version 2.6.1 [20,21]. Community prevalence rates were estimated using a hierarchical random effects model, to take into account likely local differences in presentation between clinics, using WinBUGS 1.4.1 [22].

## Results

502 cases met the inclusion criteria, and 498 had diagnoses recorded in the files. The mean age was 11 years (s.d. 3 y) and 77% were male. Three percent (16/498) of case-files recorded no disorder, 19% (94/498) emotional disorders, 5% (24/498) non-hyperactive behaviour disorders, 59% (294/498) hyperkinetic disorders (including hyperkinetic conduct disorder), 9% (47/498) mixed disorders of conduct and emotion, and 20% (98/498) other disorders. Overall, 15% (74/498) met criteria for more than one diagnostic category. The numbers of cases clinically identified as hyperkinetic disorders, concordant and discordant cases, results of DAWBA interviews, response rates and data missing at each stage in the data collection process are set out in figure 1. Of those cases who did not complete DAWBA interviews or SDQs, the clinicians responsible for the case considered contact for DAWBA interview inadvisable in 2 cases; the families refused to agree to interview in 3 cases; and the families did not attend for interview in 5 cases.

Comparing clinic diagnoses of hyperkinetic disorders against DAWBA diagnoses of ADHD identified 6 cases of DAWBA-identified ADHD not recognised by clinicians: five of these were considered to be emotional disorders; one was classified as 'other'. Only two cases of DAWBA-identified Hyperkinesis were not clinically identified: one emotional disorder and one 'other.' Clinicians only identified one case as hyperactive that the DAWBA did not detect. This child was taking stimulant medication when the DAWBA assessment was done. Overall, clinicians correctly discriminated more than 98% of cases with hyperkinetic disorders.

Among the discordant cases, clinicians significantly underdiagnosed hyperkinetic disorders relative to the DAWBA (see figure 1: 6/6 cases underdiagnosed vs. 1/26 overdiagnosed, Fisher's exact test p < 0.001) while the SDQ overidentified hyperkinetic disorders relative to clinicians: (40/328 cases overidentified vs. 2/172 underidentified, Fisher's exact test p < 0.001).

The three teams (B, M, and F) each contributed 198, 106, and 198 cases to the sample, with 11, 10, and 11 discordant cases respectively (Fisher's exact test, p = .58). As DMF was also one of the consultant psychiatrists responsible for making clinical diagnoses in one of the teams, bias could have been introduced if DMF recognised his own cases among the DAWBAs rated. However, this would have applied to DMF's team only, and in practice the non-agreed diagnoses for discordant cases were also distributed evenly between the three teams (2/11 (DMF's team), 1/10, 4/11; Fisher's exact test, p = .44).

Using cases confirmed against the study standard, the estimated median community prevalence rate for hyperkinetic disorders was 0.54% (95% interquantile range 0.23%–1.2%).

## Discussion

This study suggests that the current diagnosis of hyperkinetic disorders by UK secondary care teams is similar to that of a well-validated, standardised measure. This is markedly different to the previous research reviewed in the Introduction, and is consistent with the proposition that the introduction of governmental guidelines may have improved clinical practice in this area. While well-validated standardised measures for hyperkinetic disorders have been available for some time [23] their use in support of routine clinical diagnosis has become general in the UK only since being recommended by NICE in 2001. Similarly, in the 1980s both ICD-9 and DSM III provided detailed diagnostic criteria for hyperkinetic disorders sufficient to ensure reasonable diagnostic reliability in research settings, but which did not translate into accurate clinical practice [9] despite mounting public concern and publicity. Research published elsewhere [24] confirms that the introduction of NICE guidance was followed by an increase in the rate of treatment for hyperkinetic disorders; this paper indicates that the increase in rate was in well-diagnosed cases.

The SDQ contributed to both clinical and study diagnoses, so the study does not address the accuracy of clinic diagnoses independent of SDQ usage: this limitation was accepted as the use of validated questionnaires such as the SDQ in supporting diagnoses are specifically recommended in NICE guidance, and so are included in the current diagnostic clinical standard. Failure to use them may well contribute to underdetection [10]. The discordant cases show that, despite concerns, questionnaire cut-offs have not inappropriately replaced clinical judgement in diagnosing ADHD.

Though the confidence interval is quite wide, the estimate of community prevalence is consistent with the proportion of children with hyperkinetic disorders being referred to secondary care nationally [11], supporting the sample's representativeness.

Due both to the 2-stage design, and its inability to access school-related data for the DAWBA, the full standard was not applied to individual cases. This introduces two potential artefacts, which offer alternative explanations of the results. Firstly, the high levels of agreement in concordant cases could reflect joint over-identification by the clinician and the SDQ. This follows from the conflation of the 'possible' and 'probable' SDQ categories in defining concordant and discordant cases, as parental questionnaires' estimates are known to be approximately twice the true number of cases in the clinic setting [10,25]. Alternatively, the agreement between DAWBA and clinician in the discordant cases could be because of joint under-identification of hyperactive cases by both clinicians and the parent-only DAWBA, as Ford et al [26] found that the sensitivity of the DAWBA to ADHD was significantly reduced in the absence of school data. However, both seem unlikely. In the first case, the relatively insensitive parent-only DAWBA is both less sensitive to hyperkinetic disorders than the SDQ, and more sensitive than clinicians. It is inconceivable that clinicians could both be less sensitive to hyperkinetic disorders than the DAWBA, and also oversensitive to approximately the same extent as the SDQ. In the second, alternative case, the initial detection of "missed" hyperkinetic disorders in our study was by the SDQ, and the cutoff (at 'possible' hyperkinetic disorders) has been found to miss no cases [16,27]. While 30 of the 32 discordant cases were SDQ positive for hyperkinetic disorders in the absence of a clinical diagnosis, this total represents only 6% of the sample, and estimates by parental questionnaires such as the SDQ are known to approximately double the true number of cases in the clinic setting [10,25]. The available margin for error is thus small, and applying Ford et al's figures of a 42% reduction in sensitivity suggests that only 1–2% of the total sample is likely to have been misdiagnosed by the DAWBA for this reason. This error is very much less than that reported between clinical and standardised assessments in the studies reviewed in the introduction, and so does not invalidate the main conclusion of the study. Instead, the study found evidence of considerable SDQ oversensitivity in relation to clinician diagnosis, which would not be the case if the agreement resulted from equivalent underdetection.

Overall, the results suggest that disagreements between the DAWBA standard and clinician diagnoses are most likely to result from clinician underdetection of hyperkinetic disorders, which is consistent with previous community [11] and clinic [10] samples before or after the introduction of Government guidelines. While the very high levels of agreement between the SDQ and clinician diagnoses are greater than those found in a validation of the SDQ predictive categories [27], this can be understood by the study's use of looser clinical diagnostic criteria, using, as shown in table 1 only 4 (vs. 9) discriminatory categories to determine concordance, and the SDQ scores contributing to the clinical diagnostic process in many cases – this last being, of course, a consequence of adherence to NICE guidance.

As two teams initiated SDQ collection only if a hyperkinetic disorder was already suspected, a comparison between all three teams would reconsider Foreman et al's 2001 [10] finding that screening was needed to increase awareness of hyperkinetic disorder under the changed conditions of NICE guidelines, 4–5 years on. The lack of any significant difference between the teams is consistent with the guidance acting to appropriately increase diagnostic awareness since its introduction in 2001. Unfortunately, the study could not access closed cases, so any improvement in awareness must be inferred, rather than directly demonstrated.

## Conclusion

It seems that parents and children routinely attending secondary care clinics in the UK receive diagnoses very similar to those made using agreed, explicit standards, and so can take confidence in diagnoses of hyperkinetic disorders given to them. As this was found in services making use of governmental guidelines, the use of such guidelines should be explored in settings where similar levels of diagnostic agreement have not been achieved. A case can also be made for making structured, normed assessments like the DAWBA a routine part of the clinical assessment for hyperkinetic disorders in CAMHS, as some degree of clinician underdetection in secondary care still seems likely.

## Competing interests

Suzanne Dack was partly supported by an Unrestricted Education Grant from Lilly Pharmaceuticals (awarded to Dr David Foreman) and partly by Berkshire Mental Health NHS Trust.

David Foreman was partly supported by a Health Service Research Fellowship from the University of Reading, and partly by Berkshire Mental Health NHS Trust. Dr Foreman was also offered support by Lilly Pharmaceuticals for travel expenses to Uganda when fulfilling his role as External Examiner to Makerere Univesity.

Tamsin Ford has been supervised by Professor Robert Goodman, the originator of the DAWBA, copies of which were made available especially for this study.

No funding source had any role in the analysis and interpretation of data; in the writing of the report; and in the decision to submit the paper for publication. Berkshire Mental Health NHS Trust approved the design, and gave managerial support to data collection.

## Authors' contributions

DF initiated the study, supervised data collection, undertook the analysis and drafted the text. TF reviewed and contributed to the text and analysis.
